# Comprehensive CRISPR-Cas9 screen identifies factors which are important for plasmablast development

**DOI:** 10.3389/fimmu.2022.979606

**Published:** 2022-09-15

**Authors:** Theresa Pinter, Maria Fischer, Markus Schäfer, Michaela Fellner, Julian Jude, Johannes Zuber, Meinrad Busslinger, Miriam Wöhner

**Affiliations:** Research Institute of Molecular Pathology (IMP), Vienna Biocenter (VBC), Campus-Vienna-Biocenter 1, Vienna, Austria

**Keywords:** plasmablast, CRISPR/Cas9, sgRNA libraries, plasmablast differentiation, NIPBL, MAU2

## Abstract

Plasma cells (PCs) and their progenitors plasmablasts (PBs) are essential for the acute and long-term protection of the host against infections by providing vast levels of highly specific antibodies. Several transcription factors, like Blimp1 and Irf4, are already known to be essential for PC and PB differentiation and survival. We set out to identify additional genes, that are essential for PB development by CRISPR-Cas9 screening of 3,000 genes for the loss of PBs by employing the *in vitro*-inducible germinal center B cell (iGB) culture system and Rosa26^Cas9/+^ mice. Identified hits in the screen were *Mau2* and *Nipbl*, which are known to contribute to the loop extrusion function of the cohesin complex. Other examples of promising hits were *Taf6*, *Stat3*, *Ppp6c* and *Pgs1*. We thus provide a new set of genes, which are important for PB development.

## Introduction

The production and secretion of high-affinity antibodies is an essential part of the immune response and is the main function of plasma cells (PCs). When a mature B cell encounters an antigen, it differentiates into a short-lived, antibody-secreting plasmablast (PB) which can migrate to survival niches in the bone marrow and develop into a quiescent long-lived PC (1).

Several factors are known to be essential for PC differentiation and survival. To name some, the most prominent transcription factors are Blimp1 (2), Irf4 (3), the E-box proteins E2A and E2-2 (4), Aiolos (5) and Ikaros (6). We set out to identify additional genes essential for PB development and probed 3000 genes for their role in PB development in a Local Outlier Factor-based negative selection CRISPR screen in primary Cas9^+^ B cells which we cultured in the iGB system (7). This system allowed us to culture Cas9^+^ B cells for an extended period of time compared to other *in-vitro* PB culture systems which is necessary to evaluate loss of function effects on PB development. Newly identified targets in this screen will help us to gain a better understanding of which transcription factors directly or indirectly control PB development.

## Methods

### Mice

The *Rosa26* ^Cas9/+^ mice (8) and Blimp1^Gfp/+^ mice (9) were maintained on the C57BL/6 genetic background. All animal experiments were carried out according to valid project licenses which were approved and regularly controlled by the Austrian Veterinary Authorities.

### Antibodies for flow cytometry

The following monoclonal antibodies were used for flow cytometric analysis of the cultured cells: CD19-BV785 (1D3, BD), CD19-Fitc (1D3, Biolegend), CD19-BV421 (1D3, BD) CD23-Fitc (B3B4, ebioscience), CD43-PE (S7, Pharmingen), CD90.1-BV421 (OX-7, BD), CD138-APC (281-2, Biolegend), CD138-BV605 (281-2, BD) antibodies.

### Definition and flow cytometric sorting

The cells were sorted with a FACS Aria machine (Becton Dickinson) as follows: activated B cells (act B, CD19^+^CD23^+^CD138^–^), pre-plasmablasts (pre-PBs, CD19^+^CD23^–^CD138^–^), plasmablasts (PBs, CD19^+^CD23^–^CD138^+^).

### Selection criteria for genes tested in the screen

For the first screen we selected 998 genes, including *Irf4* and *Prdm1* as controls. 508 genes were at least 5-fold upregulated in RNA-Sequencing (RNA-Seq) data comparing either day-9 PBs (Blimp1^+^CD138^+^) and day-6 act B cells (Blimp1^–^CD138^–^) or day-9 pre-PBs (Blimp1^+^CD138^–^) and day-6 act B cells, cultured in the iGB system (7). 490 additional genes were selected as being at least 8-fold upregulated between mature B cells and PCs (10). We tested 6 single guide RNAs (sgRNAs) per gene except for some small genes where only fewer sgRNAs could be predicted.

For the second screen we targeted 2,000 genes, which were also selected from the RNA-Seq data of the iGB system. We selected genes, which had an expression of higher than 5 TPM in either the act B cells, pre-PBs or PBs. We annotated the genes and selected the categories kinases, ligand-dependent nuclear receptors, phosphatases, translation regulators, signal transducers, transcription factors, or chromatin modifiers. We then subtracted all genes which were included in the first screen or which are known to be essential for cell survival. We further excluded genes which have multiple locations in the genome. SgRNAs against *Prdm1*, *Irf4* and *Sdc1* were added as positive controls, as well as 500 neutral sgRNAs. We used 6 sgRNAs for most genes.

### RNA-sequencing

Total RNA was prepared by using the RNeasy Mini kit (QIAGEN). Genomic DNA was eliminated by using a gDNA eliminator spin column (QIAGEN). Approximately 1–5 ng of cDNA were used as starting material for the generation of sequencing libraries. About 1–5 ng of cDNA was used as starting material for the generation of sequencing libraries with the NEBNext Quick Ligation Module and NEBNext Ultra Ligation Module and NEBNext End Repair/dA-Tailing module. DNA fragments of 150–700 bp were selected with AMPure XP beads (Beckman Coulter), followed by PCR amplification with the KAPA Real Time Amplification kit (KAPA Biosystems). Completed libraries were quantified with the Agilent Bioanalyzer dsDNA 1000 assay kit and Agilent QPCR NGS Library Quantification kit. Cluster generation and sequencing was carried out through use of the Illumina HiSeq 2000 system with a read length of 50 nucleotides, according to the manufacturer’s guidelines.

### Design of sgRNA

The design of the sgRNA was performed as described before (11).

### Cloning of the library

The 81 mer oligo pools of the gene libraries were ordered from Twist Bioscience. The cloning was performed as described before (11).

### LentiX transfection

LentiX 293T cells were transfected with the library pool, using a transfection mix of the library vector pool, the helper plasmid pCMV-R8.74 and the envelope plasmid pCMV-Eco (4:2:1) by adding PEI (MW 25K, Polyscience, 24µg/ml). After 24 hours, the medium was changed to the target medium which was harvested after 12 hours and filtered (0.2 µm filters).

### Transduction of iGB-cultured B cells

The spleens of 8 week old Rosa26^Cas9/+^ mice were harvested and magnetically enriched using the B cell isolation kit (130-090-862 Miltenyi). For each sample one female and one male mouse were pooled and the cells were plated at 0.5 mio/ml in 2 ml on 6 well plates in iGB medium (RPMI medium supplemented with 10% fetal calf serum, 1 mM glutamine, 50 µM beta-mercaptoethanol, 10 mM Hepes and 1 mM sodium pyruvate), plus LPS (Sigma-Aldrich, 25 µg/ml), IL-4 (produced inhouse, 20 ng/ml), CD40L (R&D system, 100 pg/ml) and Baff (AdipoGen Life science, 100 pg/ml). After one day of culture the cells were transduced with the Lentiviral library using spinfection and Polybrene (Millipore, 5 µg/ml, 45 min, 2,400 rpm, 32°C). On day two the cells were counted and seeded on 75 cm^2^ flasks as 3 mio/flask in 25 ml iGB medium + IL-4 (20 ng/ml) + CD40L (100 pg/ml) + Baff (100 pg/ml) + G418 (Gibco, 200 µg/ml) on inactivated 40LB cells. The 40LB cells were cultured in DMEM supplemented with 10% fetal calf serum and either inactivated by 30 minutes irradiation (screen 1) or with mitomycine (Sigma-Aldrich, 10 µg/ml) which was added for 3 hours at 37°C (screen 2). 15 ml iGB medium + IL-4 (20 ng/ml) + CD40L (100 pg/ml) + Baff (100 pg/ml) +G418 (200 µg/ml) was added on day 4. On day 6 all cells were harvested, counted and reseeded or sorted. For reseeding, 3 mio cells were plated per flask in 50 flasks in 24 ml iGB medium + IL-21 (produced inhouse, 10 ng/ml) + CD40L (100 pg/ml) + Baff (100 pg/ml) + G418 (100 µg/ml). The rest of the cells was sorted for CD19^+^CD90.1(Thy1.1)^+^CD23^+^CD138^–^ (act B) and the pellet was washed with PBS and frozen at -80°C. On day 9, the cells were harvested and sorted for CD19^+^CD90.1(Thy1.1)^+^CD23^–^CD138^–^ (pre-PBs) and CD19^+^CD90(Thy1.1)^+^CD23^–^CD138^+^ (PBs). The pellets were frozen at -80°C. A representation of at least 500 cells per sgRNA was maintained for all samples.

### Library preparation for sequencing

DNA was prepared as previously described (11), as was the labeling for the sequencing (12). The PCR product was cleaned after both reactions by either AMPure XP beads (A63881, Beckman Coulter) (screen 2) or by agarose gel purification (screen 1). Sequencing was performed by the use of the Illumina HiSeq 2000 system with a read length of 50 nucleotides with 8% PhiX added, according to the manufacturer’s guidelines.

### Bioinformatical analysis

The bioinformatic analysis was done using MAGeCK (13). In the first step we calculated the guide-based DE expression, which was count based, similar to DESeq/EdgeR. We performed pairwise comparisons and calculated the log2-fold change (lfc_gene_ = mean(l2fc_gene_sgRNAs_)) and p-value. In a second step we ranked the sgRNAs based on the *P*-value (robust rank aggregation). We then tested for positive effects (elimination of gene leads to boost of cell growth) and negative effect (elimination of gene leads to cell death).

## Results

**Figure 1:**
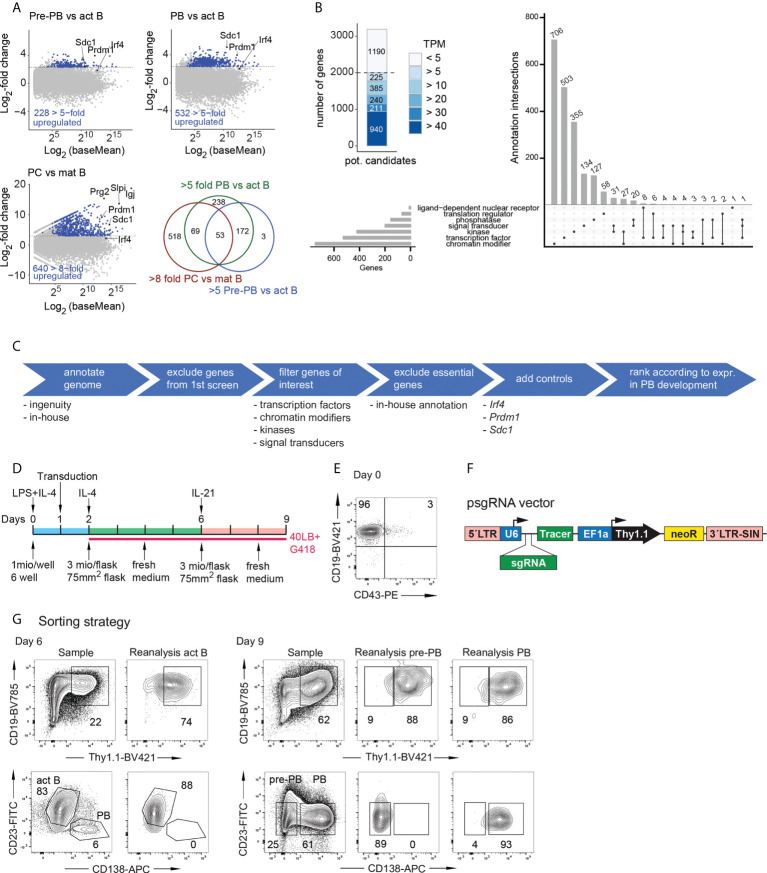
Experimental setup of the screens. **(A)** M/A blots depicting the overview of the genes which were chosen for screen 1. Data from RNA-Seq of iGB derived act B cells (CD19^+^Blimp1^–^CD138^–^), pre-PB (CD19^+^Blimp1^+^CD138^–^), or PB (CD19^+^Blimp1^+^CD138^+^) from Blimp1^Gfp/+^ mice. Chosen genes had to be at least 5-fold upregulated in the act B vs pre-PBs or act B vs PBs comparison (top row). RNA-Seq of PCs and mature B cells, depicted are genes which are 8-fold upregulated (10) (bottom row left). Overlap of the genes of the 3 M/A blots (bottom row right). **(B)** Overview of the genes of screen 2. Genes were selected by the criteria in **(C)** and grouped by their expression. Genes which did not reach an expression of at least 5 transcripts per million (TPM) in the act B, pre-PBs or PBs were discarded (left blot). The remaining genes fall into the depicted categories (Upset blot). **(D)** Timeline of the iGB culture for the screens. The cells were transduced on day 1, seeded on 40LB cells on day 2 und 6 and G418 was added from day 2 on. **(E)** Flow cytometric analysis of the splenic B cells after magnetic cell enrichment. **(F)** Structure of the psgRNA-U6-Thy1.1 vector. LTR= long terminal repeat, U6 = U6 promotor, sgRNA = single guide RNA, Tracer = tracer sequence, used for the pooled sequencing of the screen, EF1a = EF1a promoter, neoR = neomycin resistance. **(G)** Flow cytometric sorting of the cells of screen 2 on day 6 (left) or day 9 (right). The unsorted sample is shown and the reanalysis of the different populations after the cell sort.

To identify novel factors required for PC or PB development and survival, we performed two separate CRISPR-Cas9 screens in the iGB system with two distinct PB/PC transcriptome based libraries. This divided approach was used because targeting everything in one approach would not have been experimentally feasible. To enrich the library for potentially relevant genes, we employed the following transcriptomics based strategy: Screen 1 contained sgRNAs targeting 998 genes of which 508 are 5-fold upregulated either between act B and pre-PBs or between act B and PBs in the iGB system (**Figure 1A**). Another 480 genes were at least 8-fold upregulated between mature B cells and PCs (10). By including genes that are primarily expressed in PBs and their precursors, we expected to target factors that are truly relevant to PB development and not earlier in the B cell lineage. Screen 2 targeted 2000 selected genes, which are expressed at a TPM of at least 5 in either act B, pre-PBs or PBs in the iGB (**Figure 1B**) and belong to selected gene categories (**Figures 1B, C**). This approach allowed us to test genes which are expressed in late B cell development regardless of whether they show any difference in their expression. They might, nevertheless, play a more subtle role in PB development. SgRNAs against *Prdm1* (Blimp1)*, Irf4* and *Sdc1* (CD138) served as positive controls in both screens.

To enable ‘near-physiological’ B cell differentiation *in vitro*, we used the iGB system, in which B cells from the spleen are cultured on 40LB feeder cells which express CD40L and Baff for up to 10 days (7). As B cells are rather difficult to transduce, we modified the culture to efficiently achieve high transduction rates in the following way (**Figure 1D**). First, B cells from *Rosa26* ^Cas9/+^ mice (8) were isolated by magnetic cell sorting (**Figure 1E**) and plated in iGB medium containing IL-4, CD40L, Baff and LPS and were kept in culture for 24 hours. This activation of B cells increases transduction efficiency. Then the cells were transduced using ecotropic envelope packaged lentiviral particles (**Figure 1F**). After another 24 hours the cells were plated on the 40LB feeder cells in iGB medium containing G418. On day 6 and 9 of the culture the cells were sorted for day-6 act B cells (CD19^+^ Thy1.1^+^ CD23^+^ CD138^–^), day-9 pre-PBs (CD19^+^Thy1.1^+^CD23^–^CD138^–^) and day-9 PBs (CD19^+^Thy1.1^+^CD23^–^CD138^+^) (**Figure 1G**). Considering the timeframe of 9 days, we were not only able to efficiently delete a given gene of interest in act B cells but also to look at subsequent effects on PB development.

**Figure 2:**
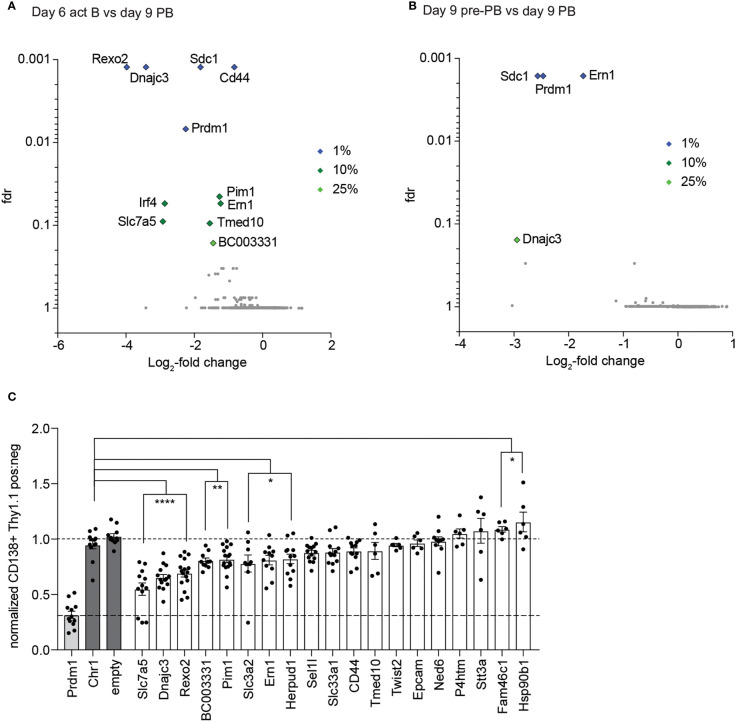
Results of screen 1. **(A, B)** Results of screen 1. Volcano blots, depicting the negative enrichment of sgRNAs, from the comparison act B vs PBs **(A)** and pre-PBs vs PBs **(B)**. Fdr= false discovery rate. **(C)** Single guide validation of first screen. The ratio of the amount of PBs in the Thy1.1 negative and positive population was calculated and normalized to the average of the value of the negative controls Chr1 (targeting a gene free region on Chromosome 1) and empty vector (the plasmid without sgRNAs). Top two performing sgRNAs from the screen were used for each gene. Each dot represents a replicate. All statistical data show as mean values with SEM; *P < 0.05; **P < 0.01; ****P < 0.0001 (Student’s *t* test).

For screen 1 we obtained data from four replicates of the act B cells and pre-PBs and three replicates of the PBs. When we compared the act B cells with the PBs, we found sgRNAs targeting 5 genes which were significantly depleted in the PB population with a false discovery rate (fdr) of at least 1% (**Figure 2A**; **Supplementary Table 1**). Besides our positive controls *Prdm1* and *Sdc1*, those were *Rexo2, Dnajc3* and *Cd44*. Another 5 genes had a fdr of at least 10% (*Slc7a5, Pim1, Ern1, Tmed10* and *Irf4)* and 1 gene, *BC003331*, had a fdr of less than 25%. Besides the positive controls *Prdm1* and *Sdc1*, sgRNAs targeting *Ern1* and *Dnajc3* resulted in a depletion of PBs compared to pre-PB (**Figure 2B**; **Supplementary Table 1**). Based on the effect size of the screen we selected 19 genes for single knockout validations in the iGB system. To score the effect of each knockout on PB differentiation capacity, we compared Thy1^+^ with Thy1^–^ PBs. To correct for PB formation capacity of infected versus non-infected Rosa26^Cas9/+^ cells, this ratio was normalized to the neutral control knockouts empty (empty vector) and Chr1 (a sgRNA targeting a gene desert region on chromosome 1). As a positive control, samples were transduced with sgRNAs targeting *Prdm1*. We could confirm *Slc7a5, Dnajc3, Rexo2, BC003331, Pim1, Slc3a2, Ern1* and *Herpud1* as being important for PB development or PB survival (**Figure 2C**).

**Figure 3:**
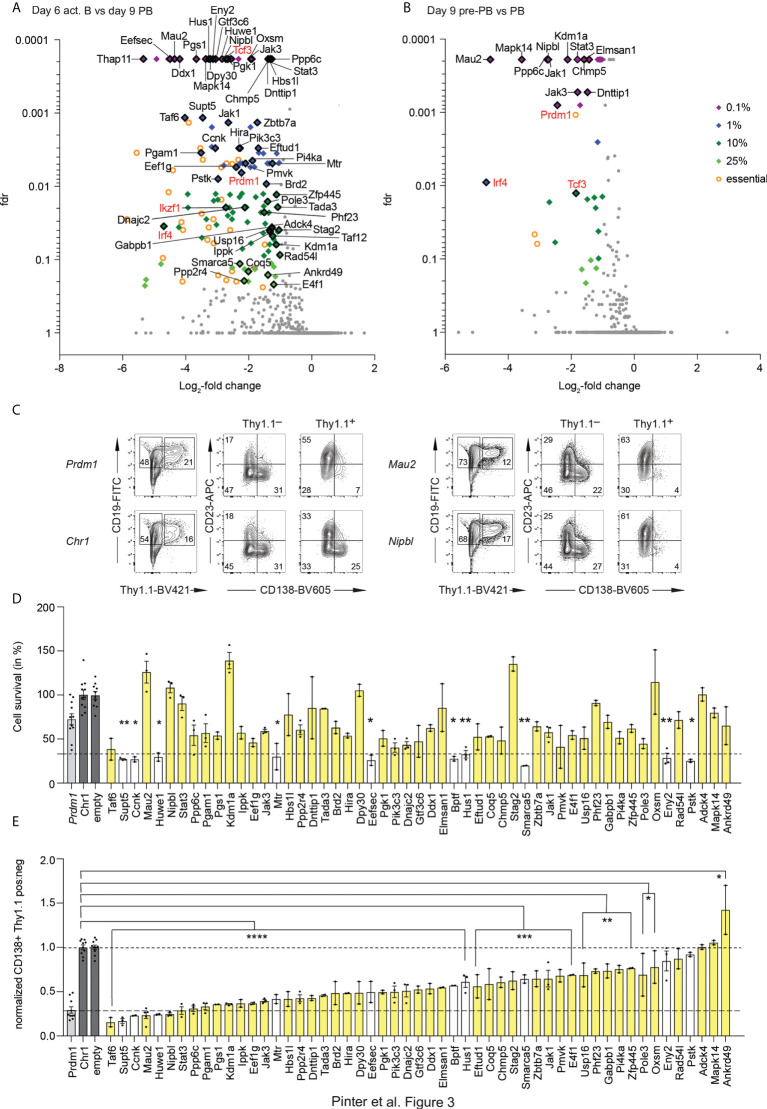
Results of screen 2. **(A, B)** Volcano blot of the screen 2, depicting interesting hits in the comparison of the act B from day 6 versus the PBs from day 9 **(A)** and the pre-PBs versus the PBs from day 9 **(B)**, as in **Figure 1G**. Fdr = false discovery rate. Factors which are known to be essential for PCs are highlighted in red. **(C)** Example of single guide validation. Control Chr1 is targeting a gene free region on Chromosome 1. **(D, E)** Single guide validation. Cell survival was determined by the ratio of the amount of Thy1.1^+^ cells on day 6 versus day 9, normalized to Chr1 and the empty vector (the plasmid without any sgRNAs). Stars indicate significant difference compared to Chr1 **(D)**. The ratio of the amount of PBs in the Thy1.1 negative and positive population was calculated and normalized to the average of the value of the negative controls Chr1 and empty vector to determine the impact of the sgRNA on the PBs **(E)**. One or two best performing single sgRNAs from the screen were used. Each dot represents one replicate. Genes colored in white showed reduced survival in **(D)**. All statistical data are shown as mean values with SEM; *P < 0.05; **P < 0.01; ***P < 0.001; ****P < 0.0001 (Student’s *t* test).

Screen 2, which contained sgRNAs targeting 2000 genes that are expressed in act B cells, pre-PBs and PBs, was performed and analyzed in a similar manner as screen 1. We obtained 2 replicates of independent iGB cultures each of the 6 days act B cells, 9 days pre-PBs and PBs with a representation of at least 500 cells per sgRNA. We also sequenced the sgRNA pool used for transduction of the cells. To remove genes which were essential for all tested cell populations, we first compared the initial pool of sgRNAs with the act B cells and defined genes which scored in this comparison with a log-fold change (lfc) of smaller than -1 and a fdr of less than 1% as essential. The remaining genes were selected for a lfc < -1 and sorted by their fdr of the comparison act B versus PBs to identify the most promising hits. For screen 2, this revealed 26 genes with a fdr < 0.1%, 30 genes with a fdr < 1%, 48 genes with a fdr < 10% and 15 genes with a fdr < 25% (**Figure 3A**; **Supplementary Table 2**). Our controls *Prdm1* and *Irf4* scored in this analysis, as did *Ikzf1* (Ikaros) and *Tcf3* (E2A), which have also been shown to be essential for PCs (4, 6). Similarly, we compared the initial pool of sgRNAs with the pre-PBs and defined genes which scored in this comparison with a lfc < -1 and a fdr of less than 1% as essential. We identified the most promising hits in the comparison pre-PBs with PBs and found 20 genes with a fdr < 0.1%, 2 genes with a fdr < 1%, 9 genes with a fdr < 10% and 4 genes with a fdr < 25% (**Figure 3B**; **Supplementary Table 2**). We validated 53 of the hits from both analyses in single validations using the 2 best performing sgRNAs from the screen (**Figures 3C–E**). We looked at 2 criteria. First, whether the cell survival of the transduced cells was impaired, by comparing the amount of transduced cells on day 6 and day 9 (**Figures 3C, D**) and second whether the PBs in the transduced cell population were reduced (**Figures 3C, E**). 39 genes showed a reduction in PBs without impairing the survival of the cells. The high confidence hits with the biggest impact specifically on PBs were *Mau2*, *Nipbl, Stat3, Ppp6c*, *Pgam1, Pgs1, Kdm1a, Ippk, Eef1g, Jak3, Hbs1l, Ppp2r4, Dnttip1, Tada3, Brd2, Hira, Dpy30, Pgk1, Pik3c3, Dnajc2, Gtf3c6, Ddx1* and *Elmsan1*.

## Discussion

In this study, we screened 3,000 genes for their impact on PB development and survival. We expected to find new promising genes in the first screen where 1000 genes, which are upregulated in the PBs or PCs, were targeted. Strongly upregulated genes are often expected to be important for the respective cell type, but surprisingly the number of genes of which the depletion impacted the PBs was rather small. One of the top hits from this screen were the genes *Slc7a5* and *Slc3a2*, which together form CD98. It has been shown that the loss of CD98 leads to strongly reduced PC numbers (14), consistent with our finding. The second screen in which 2000 genes of different important categories which are at least moderately expressed in the PBs were screened, revealed a vaster number of interesting genes. 24 of those genes can be related to biosynthetic pathways (15), which is in line with the massive metabolic changes PBs undergo in order to maintain a high antibody secretion (16) and are thus sensitive to the loss of genes involved in metabolism. However, the genes in the second screen were preselected for certain categories, making a functional enrichment analysis rather biased. The hits in this screen also included the genes *Nipbl* and *Mau2*, which are known to contribute to the loop extrusion function of the cohesin complex (17, 18). Those factors have never been shown to be specifically important for PBs. Another top scoring candidate gene, *Kdm1a* (Histone demethylase LSD1), has been shown to regulate B cell proliferation and PB differentiation which further validates the validity of our approach (19). In total we could confirm 8 hits from the screen 1 and 39 hits from the screen 2 to be essential for PBs without affecting the overall cell survival in the iGB system, which means that we have identified 47 hits to be exclusively relevant to PB development. An important step for the future would be an *in vivo* validation of those hits.

One advantage of our culture system over other common B cell cultures, like LPS stimulation, was the culture time. Act B cells were cultured for 6 days before we induced PB development. As the cells were infected on day 1 of the culture with the sgRNA library to induce gene inactivation, loss of the respective proteins from the cells should be complete at day 5 after infection. Hence by the time PB development was induced, the cells were full knockouts of the targeted genes.

A similar screen was recently published (20). While our approach followed the same principle, there are numerous differences. The screen of Newman et al. (2021) largely focused on the difference between IgG1^+^ versus IgE^+^ PBs, while we compared different developmental stages (act B cells, pre-PBs versus PBs). Our screen thus focuses on the development that leads to the PB formation while theirs looks at differences when the cell are already PBs. There are further differences, like the screened sgRNA library itself. Newman et al. used an untailored sgRNA library, which allows them on one hand to find unexpected targets, but on the other hand, includes a lot of sgRNAs targeted against genes which are not expressed in PBs at all. Our sgRNA library was tailored for the iGB cells and contained all genes, which are upregulated between the act B cells and the PBs or between mature B cells and PCs and another 2,000 genes which are expressed in those cell types and fall into categories of interest.

With our approach we managed to target 3,000 genes and thus supply a new and extensive list of genes that are important for the development or survival of plasmablasts.

## Data availability statement

The datasets presented in this study can be found in online repositories. The names of the repository/repositories and accession number(s) can be found below: https://www.ncbi.nlm.nih.gov/geo/, GSE203436.

## Ethics statement

The animal study was reviewed and approved by Austrian Veterinary Authorities. Written informed consent was obtained from the owners for the participation of their animals in this study.

## Author contributions

TP performed the single guide validation of the second screen, MaF performed the bioinformatic analysis, MiF cloned the sgRNA library, JJ and MS provided assistance and knowledge for the design of the project and the preparation of the DNA for the sequencing, JZ provided the tools for the sgRNA prediction and the library plasmid, MB and MW planned the project and MW performed the screens, the single guide validation of the first screen and wrote the manuscript. All authors contributed to the article and approved the submitted version.

## Funding

The authors declare that this study received funding from Boehringer Ingelheim. The funder was not involved in the study design, collection, analysis, interpretation of data, the writing of this article, or the decision to submit it for publication. This research was also supported by the European Research Council (ERC) under the European Union’s Horizon 2020 research and innovation program (grant agreement No 740349- PlasmaCellControl) and the Austrian Research Promotion Agency (Early Stage Grant ‘Molecular Control’ FFG-878286). TP was supported by a Boehringer Ingelheim Fonds PhD fellowship. MS is a member of the Boehringer Ingelheim Discovery Research global post-doc program.

## Acknowledgments

We thank G. Schmauß and M. Weninger for FACS sorting, A. Sommer and his team at Vienna BioCenter Core Facilities (VBCF) for Illumina sequencing and H. Clevers for providing the *Rosa26* ^Cas9/+^ mice.

## Conflict of interest

The authors declare that the research was conducted in the absence of any commercial or financial relationships that could be construed as a potential conflict of interest.

## Publisher’s note

All claims expressed in this article are solely those of the authors and do not necessarily represent those of their affiliated organizations, or those of the publisher, the editors and the reviewers. Any product that may be evaluated in this article, or claim that may be made by its manufacturer, is not guaranteed or endorsed by the publisher.

## References

[1] Nutt SL Hodgkin PD Tarlinton DM Corcoran LM . The generation of antibody-secreting plasma cells. Nat Rev Immunol (2015) 15:160–71. doi: 10.1038/nri3795 25698678

[2] Shaffer AL Lin KI Kuo TC Yu X Hurt EM Rosenwald A . Blimp-1 orchestrates plasma cell differentiation by extinguishing the mature b cell gene expression program. Immunity (2002) 17:51–62. doi: 10.1016/S1074-7613(02)00335-7 12150891

[3] Mittrücker HW Matsuyama T Grossman A Kündig TM Potter J Shahinian A . Requirement for the transcription factor LSIRF/IRF4 for mature b and T lymphocyte function. Science (1997) 275:540–3. doi: 10.1126/science.275.5299.540 8999800

[4] Wöhner M Tagoh H Bilic I Jaritz M Poliakova DK Fischer M . Molecular functions of the transcription factors E2A and E2-2 in controlling germinal center b cell and plasma cell development. J Exp Med (2016) 213:1201–21. doi: 10.1084/jem.20152002 PMC4925024 27261530

[5] Cortés M Georgopoulos K . Aiolos is required for the generation of high affinity bone marrow plasma cells responsible for long-term immunity. J Exp Med (2004) 199:209–19. doi: 10.1084/jem.20031571 PMC2211773 14718515

[6] Schwickert TA Tagoh H Schindler K Fischer M Jaritz M Busslinger M . Ikaros prevents autoimmunity by controlling anergy and toll-like receptor signaling in b cells. Nat Immunol (2019) 20:1517–29. doi: 10.1038/s41590-019-0490-2 PMC7115902 31591571

[7] Nojima T Haniuda K Moutai T Matsudaira M Mizokawa S Shiratori I . *in-vitro* derived germinal centre b cells differentially generate memory b or plasma cells. vivo Nat Commun (2011) 2:465. doi: 10.1038/ncomms1475 21897376

[8] Gehart H van Es JH Hamer K Beumer J Kretzschmar K Dekkers JF . Identification of enteroendocrine regulators by real-time single-cell differentiation mapping. Cell (2019) 176:1158–73.e16. doi: 10.1016/j.cell.2018.12.029 30712869

[9] Kallies A Hasbold J Tarlinton DM Dietrich W Corcoran LM Hodgkin PD . Plasma cell ontogeny defined by quantitative changes in blimp-1 expression. J Exp Med (2004) 200:967–77. doi: 10.1084/jem.20040973 PMC2211847 15492122

[10] Minnich M Tagoh H Bönelt P Axelsson E Fischer M Cebolla B . Multifunctional role of the transcription factor blimp-1 in coordinating plasma cell differentiation. Nat Immunol (2016) 17:331–43. doi: 10.1038/ni.3349 PMC5790184 26779602

[11] Michlits G Jude J Hinterndorfer M de Almeida M Vainorius G Hubmann M . Multilayered VBC score predicts sgRNAs that efficiently generate loss-of-function alleles. Nat Methods (2020) 17:708–16. doi: 10.1038/s41592-020-0850-8 32514112

[12] de Almeida M Hinterndorfer M Brunner H Grishkovskaya I Singh K Schleiffer A . AKIRIN2 controls the nuclear import of proteasomes in vertebrates. Nature (2021) 599:491–6. doi: 10.1038/s41586-021-04035-8 34711951

[13] Li W Xu H Xiao T Cong L Love MI Zhang F . MAGeCK enables robust identification of essential genes from genome-scale CRISPR/Cas9 knockout screens. Genome Biol (2014) 15:554. doi: 10.1186/s13059-014-0554-4 25476604 PMC4290824

[14] Cantor J Browne CD Ruppert R Féral CC Fässler R Rickert RC . CD98hc facilitates b cell proliferation and adaptive humoral immunity. Nat Immunol (2009) 10:412–9. doi: 10.1038/ni.1712 PMC2672195 19270713

[15] Liao Y Wang J Jaehnig EJ Shi Z Zhang B . WebGestalt 2019: gene set analysis toolkit with revamped UIs and APIs. Nucleic Acids Res 47 W199–W205 (2019) 47(W1):W199–W205. doi: 10.1093/nar/gkz401 PMC6602449 31114916

[16] Boothby M Rickert RC . Metabolic regulation of the immune humoral response. Immunity (2017) 46:743–55. doi: 10.1016/j.immuni.2017.04.009 PMC5640164 28514675

[17] Ström L Lindroos HB Shirahige K Sjögren C . Postreplicative recruitment of cohesin to double-strand breaks is required for DNA repair. Mol Cell (2004) 16:1003–15. doi: 10.1016/j.molcel.2004.11.026 15610742

[18] Unal E Arbel-Eden A Sattler U Shroff R Lichten M Haber JE . DNA Damage response pathway uses histone modification to assemble a double-strand break-specific cohesin domain. Mol Cell (2004) 16:991–1002. doi: 10.1016/j.molcel.2004.11.027 15610741

[19] Haines RR Barwick BG Scharer CD Majumder P Randall TD Boss JM . The histone demethylase LSD1 regulates b cell proliferation and plasmablast differentiation. J Immunol (2018) 201:2799–811. doi: 10.4049/jimmunol.1800952 PMC6200601 30232138

[20] Newman R Tolar P . Chronic calcium signaling in IgE+ b cells limits plasma cell differentiation and survival. Immunity (2021) 54:2756–71.e10. doi: 10.1016/j.immuni.2021.11.006 34879220

